# The Role of Plant-Based Protein Functional Food in Preventing Acute Respiratory Disease: A Case Study

**DOI:** 10.3390/nu13062116

**Published:** 2021-06-20

**Authors:** Andrei V. Tarasov, Rofail S. Rakhmanov, Elena S. Bogomolova, Ludmila A. Perminova, Zhanna L. Malakhova

**Affiliations:** 1 Department of Pediatrics and Preventive Medicine, Medical Institute, Immanuel Kant Baltic Federal University, 14 A. Nevskogo ul., 236016 Kaliningrad, Russia; perminova72@mail.ru (L.A.P.); me-to-you1@yandex.ru (Z.L.M.); 2 Department of hygiene, Privolzhsky Research Medical University, 10/1, Minin and Pozharsky Sq., 603950 Nizhniy Novgorod, Russia; raf53@mail.ru (R.S.R.); olenabgm@rambler.ru (E.S.B.)

**Keywords:** students, social adaptation, acclimatization, functional food, prevention, respiratory diseases, immunity, blood test, vitamins, antioxidants

## Abstract

The Kaliningrad region is known for its specific climate, which can negatively affect the adaptive potential of the body. This manifests in an increased incidence of respiratory diseases and skin conditions. To prevent high morbidity, a plant protein product was included in the diet of first-year university students. This study aimed to assess the effectiveness of this food intervention in preventing the most common diseases among Kaliningrad students. Two groups of university students took part in the food trial. In the control group, catabolic processes prevailed in nutrient metabolism. Disadaptation manifested itself in the metabolism of proteins, vitamins, minerals, hematopoiesis and humoral immunity. Inflammation was indicated by α1- and α2-globulins, a weak immune response, and IgM and IgG. High oxidative stress and low antioxidative ability of blood serum were observed. The plant-based protein product (FP) helped preserve testosterone level and prevent an increase in catabolic reactions. Moreover, it had a positive effect on both red blood cell hematopoiesis (a smaller increase in the average volume of erythrocytes, the same average concentration and content of hemoglobin, an increased relative red cell distribution width (RDW) and white blood cell hematopoiesis (a beneficial effect for the immune system: lymphocytes, the relative content of neutrophils, monocytes, basophils and eosinophils). The stimulation of humoral immunity was evidenced by beta- and gamma-globulins, an active immune response, the level of IgM and IgG, antioxidant protection, reduction of peroxides and an increase in antioxidant activity of blood serum. The 34-week observation showed a 1.7-fold decrease in the incidence of respiratory illnesses and a 5.7-fold decrease in skin and subcutaneous tissue diseases. Acute respiratory infections were reduced 1.8-fold. There were no cases of community-acquired pneumonia in the treatment group, compared with 55.1‰ in the control group. The incidence of respiratory diseases was 3.3–10.6 times lower in the treatment group than in the control group in weeks 6–19. The findings testify to the prophylactic effect of functional food during social adaptation and acclimatization of students.

## 1. Introduction

Based on recent advances in science and technology, modern university education shows a steady tendency towards an increase in the study load and, consequently, in a higher level of stress [1,2,3,4], considerable disruption of habitual daily routines [5,6,7], irregular meals [8,9,10,11,12,13], decreased motor activity [14,15,16,17], weaker physiological functions of the body and other health risks [18,19,20,21]. During the first years after their relocation to the place of study, students’ health can be affected by many factors, first and foremost by a much higher study load in university compared with school, a different organization of the study process and the need for social and professional adaptation [22,23,24,25]. Consequently, the development and introduction of new methods of preventing diseases and reducing health risks require further research [26,27]. Upon entering into university, many students have to move to another region, often with different weather and climate conditions. This poses a health risk for students who need to adapt to a new type of climate. The climate of the Kaliningrad region has its specific seasonal variations, which have become substantial in recent decades [28]. In our previous works, we showed that the climate of the region is clinically unfavorable, which is evidenced by an increased incidence of respiratory and skin diseases amongst incoming students. Hence, there is a need for more efficient methods of disease prevention in this social group [29].

It is now known that the main reason for the growth of chronic non-infectious diseases—a major cause of morbidity and mortality in many regions of Russia—is the deficit of macro- and micronutrients in the diet. A poor and unbalanced diet leads to disorders of the immune system and a decrease in the body’s resilience to negative environmental factors [30]. For instance, the quarantine imposed during the COVID-19/SARS-CoV-2 pandemic is strongly associated with stress and depression, which lead to unhealthy diets and reduced physical activity. A diet low in fruit and vegetables is common during self-isolation, resulting in a low intake of antioxidants and vitamins. It is noteworthy that vitamins have been one of the main “weapons” in the fight against COVID-19/SARS-CoV-2 [31]. The spread of COVID-19 and its high mortality rate could be related (among other factors) to an unhealthy lifestyle, which leads to a low-grade inflammation (LGI) that increases not only the risk of chronic diseases but also the risk of complications relating to infections and greater susceptibility to infections. Recent research has demonstrated that the microbiota plays a fundamental role in modulating metabolic responses in the immune system. There is a correlation between lifestyle, the state of the immune system, and infections. The immune response is compromised if nutrition is unbalanced or insufficient, because daily diet affects the intestinal flora and can make individuals more susceptible to infections. The nutritional state can also be aggravated by the immune response to the infection [32]. In six countries (France, Germany, Italy, Spain, UK, USA), plant-based diets or pescatarian diets were associated with lower odds of moderate-to-severe COVID-19. These dietary patterns may be considered for protection against severe COVID-19 [33].

Since the 1990s, functional foods have become an integral part of the diet in many countries. Functional foods are known to be highly nutritious and beneficial for physical and mental health. Functional food products have an increased content of biologically active ingredients, which can prevent the development of pre-nosological conditions. The term *pre-nosological condition* first appeared in the Medical Encyclopaedia in 1978 [34]. It has been widely used in medicine and physiology ever since. The transition from health to illness is not instant. There is a spectrum of intermediate conditions between the two states. Thus, the term *pre-nosological conditions* refers to a state when regulatory systems and functions of the body are strained to a greater degree than normal to sustain life [35]. 

There is an extensive body of literature describing the efficacy of functional foods in preventing diseases in different risk groups, including students and schoolchildren [36,37,38,39,40,41]. The term *functional food* first appeared in Japan, which is the world leader in the development of food for special health issues (FOSHU) [42]. 

It has to be noted that there is no universally accepted definition of what functional food is. Generally speaking, all foods are functional since they are active at a particular physiological level. The American Dietetic Association (ADA) defined functional food as “wholesome food and enriched food products having a potentially beneficial effect on human health when consumed regularly and in the right amounts” [43]. In Russia, the definition of *functional product* (FP) is given in the state standard P 52349-2005 and reads as “a food product designed for systematic use as part of a diet of all age groups of healthy people; it reduces the risk of diet-related diseases and improves health by means of physiologically active ingredients used in combination” [44]. The use of FP for improving the health of different population groups in Russia is regulated by the Decree of the Government of the Russian Federation of 25.10.2010 № 1873 “Fundamentals of the State Policy in the Field of Health Nutrition for the Period up to 2020”. The decree has separate provisions on the development and production of functional foods [45].

This study aims to analyze the effectiveness of a functional food product enriched with macro- and micronutrients as a means of restoring the vitamin and mineral balance of the body thus reducing morbidity among students during their social adaptation and acclimatization.

## 2. Materials and Methods

### 2.1. Preparation of the Functional Food Product

In our study, we used an FP manufactured using cryogenic technology. Freezing facilitates several processes. Firstly, it enables the cutting of raw materials that cannot be handled using any other treatment method due to changes in mechanical properties (embrittlement). Secondly, it allows powdering to a particle size that cannot be obtained at higher temperatures (fine and extra-fine powdering). Thirdly, it is the preservation of biologically active elements in the ground product (the ingredients are not heated, and they are in an inert atmosphere). Fourthly, it allows the prevention of particle aggregation. This technique makes it possible to increase the bioavailability of active elements to the body. This is a greener method of production compared with alternative methods that use chemical processing [46]. 

Cryogenic technology makes it possible to preserve the outer layer of plant seeds, as well as to avoid blanching and sulphitization, and perform drying and milling operations at extremely low temperatures [47,48]. The manufacturing of FP usually involves several stages. It starts with a primary preparation of fresh raw materials (sorting, washing, removal of foreign inclusions, etc.). The first production stage is sublimation—the removal of water content from frozen raw materials at a temperature from −10 °C to −30 °C. This allows manufacturers to fully preserve all biologically active elements of plants already at the stage of drying (moisture removal). The second stage is mechanical crushing, performed at a temperature of −110 °C, which is followed by cryogenic crushing at a temperature of −196 °C to a particle size of 2.5–160 microns. 

Raw materials were ground using the Plasma-10NS cryo-grinder in the presence of liquid nitrogen for one hour. Then, cryopowders were sieved using a vibrating nylon sieve with a mesh diameter of 0.6 mm and weighed on SVP 50-5 scales with an accuracy of 0.1 g. Powders were packed in metalized stand-up pouches and plastic bottles with opening control. Synthetic substances were not used in the manufacturing of the products. The pills did not contain colorant agents, flavors, preservatives or other chemicals [49]. End products were finely dispersed cryopowders, with a high content of biologically active elements. The consumption of one gram of a cryopowder equals the consumption of 700–1000 g of fresh fruits or vegetables. Drying makes it possible to increase the concentration of biologically active substance 8.0–12.0-fold by moisture removal (raw materials contain up to 90.0% of water before drying and 5.0–10.0% after it). Milling prevents raw material oxidation and releases biologically active elements bound to protein molecules. This way, the elements are fully absorbed by the human body. Recent studies have shown the benefits of this technology: during tests described in the literature, the content of carotenoids in wild ashberry increased 1.84 times; of thiamine, 16.0 times; of vitamin E, 10.0 times. In cranberry, the content of thiamine rose 11.0 times, and that of riboflavin 12.0 times [46].

The removal of water content from vegetables and fruits significantly increases the extraction properties of FPs and the degree of their assimilation [50,51].

### 2.2. The Composition of the Functional Product

Many types of functional food have a high vitamin and mineral content. Vitamin deficiency negatively affects human health, reduces performance and weakens resistance to infectious diseases and changes in the environment. Prolonged vitamin deficiency in adolescence adversely affects overall physical condition and stamina and leads to metabolic disorders and chronic diseases. The most common vitamin deficiency is that of vitamins A, B_1_, B_2_ [52]. 

Minerals are inorganic components of food, which are important for maintaining health. Their deficiency or excess may lead to serious consequences. The most important mineral macronutrients are calcium, phosphorus, sodium, potassium and magnesium, and the most essential microelements are iron, iodine, zinc, copper, and fluoride. In adolescents, calcium, iron and zinc are an integral part of their daily diet for their healthy physical development. The main natural sources of vitamins and minerals are vegetables, fruits and berries [52].

The composition of a functional food product can affect the morpho-functional condition of humans by restoring the vitamin and mineral balance. Here, it is essential to consider what doses of vitamins and minerals per kg of body weight stimulate the metabolic processes, reduce adaptation stress and increase nonspecific resistance [53]. Based on the above, an FP was selected as a food intervention to redress the vitamin and mineral imbalance in students. The FP had a high vitamin (A, B_1_, B_2_, E, K_1_, C) and mineral content (Cu, Zn, Fe, Mn, Cr) and was designed to have a beneficial effect on the immune system. 

Vitamin A is a potent antioxidant; it is involved in the completion of phase III and IV of phagocytosis and increases the synthesis of IgE and IgG. It stimulates the formation of T-killers in stem lymphocytic cells and Th2-helpers by increasing the production of IL-4 and IL-5. Vitamin A deficiency leads to a decrease in the level of B-lymphocytes and T-helpers. Vitamin A is involved in redox reactions and protein synthesis, promotes normal metabolism, improves the function of the cell and subcellular membranes and plays an important role in the formation of bones and teeth. It is essential for the growth of new cells and slows down aging. Vitamin E is involved in the production of cytokines. It suppresses the generation of antigen-specific and antigen-specific suppressors and thus stimulates cellular and humoral immune responses. It has a positive effect on the function of macrophages, and it is a strong antioxidant. Vitamin B_2_ is a cofactor in the activity of glutathione reductase in erythrocytes and leukocytes. Riboflavin is essential for the formation of red blood cells and antibodies, for cell respiration and growth. It facilitates the absorption of oxygen by the cells of the skin, nails and hair [54]. 

Copper increases the activity of macrophages. With a deficiency, there is a decrease in CD4 (T-helpers) and suppression of the functional activity of T- and B-lymphocytes. It participates in enzymatic catalysis (biocatalysis) and electron transfer and interacts with iron. Copper deficiency leads to leukopenia and neutropenia. Copper has a pronounced anti-inflammatory property and mitigates the manifestation of autoimmune diseases. Iron is part of transferrin, which is a lymphocyte activator. It has been established that low iron content in the body weakens the function of the immune system such that the saturation of tissues with granulocytes and macrophages decreases. Phagocytosis, the response of lymphocytes to stimulation with antigens, and the formation of antibodies are inhibited. A decrease in the level of iron in the body causes a sharp inhibition of the cytotoxic function of killer cells, and, along with this, the production of interferon by macrophages decreases [55].

Phosphorus has a crucial role in the acid-base equilibrium of blood. As part of many organic compounds, it is involved in metabolism. Phosphorous deficiency is associated with general and calcium metabolism disorders, rickets and osteomalacia. Magnesium participates in bone tissue and tooth development, neuromuscular conduction, and ATP-dependent and kinase reactions. It is a coenzyme in carbohydrate and protein metabolism and an essential component of intracellular fluid. Magnesium compounds activate enzymes, particularly those taking part in calcium and phosphoric metabolism [54].

The FPs contained the following ingredients: watermelon seeds (13%), oats (8%), rose hips (20%), spinach (8%), kelp (16%), parsley (15%), celery (10%) and egg white (10%). The product was used in pill form. The composition of the functional product was determined based on an assessment of vitamins and minerals used in different proportions. The doses of vitamins (A, E) and minerals (copper, zinc, iron) were identified by analyzing metabolic processes in the body [53]. This FP has been registered and authorized for sale in the Eurasian Economic Union [56].

### 2.3. The Selection of Groups of Students and Methods of Blood Sample Analysis

The aim of our study was to evaluate the efficiency of student diet optimization by adding a functional food product with a high concentration of biologically active elements. The evaluation of the efficiency of the proposed product was based on the assessment of the metabolic status and morbidity. We carried out a randomized controlled study into the impact of the Baltic climate on the health of students. More specifically, the object of research was the efficiency of a plant-based protein functional product in preventing common diseases included in the International Classification of Diseases (ICD-10). To do this, we studied two groups of first-year students who had come to Kaliningrad from other Russian regions. There were 50 students in each group. All students gave their informed voluntary consent to participate in the study. Students were randomly assigned to treatment and control groups. The study was conducted with the permission and under the supervision of the Ethics Committee of Privolzhsky Medical Research University. All students were healthy and underwent a medical examination before entering into the trial. Although the diet of the two groups was balanced and the same (organized catering), the students’ adaptation to a new social environment and simultaneous acclimatisation caused an increased uptake of vitamins and minerals, which led to disadaptation changes in the body [57]. This conclusion led us to optimize the diet by including a functional product rich in biologically active elements. Compared with the control group, the diet of the treatment group also included 0.9 g of the functional product (FP) given twice a day (during breakfast and dinner) for 15 days. The FP was given 4–5 weeks after the arrival of students in the region.

The concentration of vitamins and minerals in the FP, as well as in blood and blood serum, was determined by the Research Institute of Hygiene and Occupational Pathology of Rospotrebnadzor (Nizhny Novgorod). We determined the content of vitamins A (retinol and carotenoids), E (alpha-tocopherol) and K (K_1_) in the FP. The content of vitamin A (retinol) and vitamin E (alpha-tocopherol) was determined in blood serum and that of vitamin B_2_ (riboflavin) in whole blood. The content of the decomposition product of pyruvic acid (PVA) indicates the saturation of the body with vitamin B_1_ (a higher level is an indicator of a decrease in the vitamin supply of the body). The concentration of minerals was measured in blood serum. 

Blood samples were taken three times: after a meal including the FP (15 days into the observation) and after another 30 days (45 days into the observation). Blood samples were taken from the cubital vein after overnight fasting. Blood was collected with a vacuum blood collection tube with heparin. Within one hour, samples were delivered to a clinical laboratory where a general blood test was performed. 

Blood serum and whole blood samples were refrigerated and sent to Nizhny Novgorod (the Institute of Hygiene and Occupational Pathology) following the protocols for the transportation of frozen biomaterials. Samples included total protein and protein fractions (albumin, α1-, α2-, β- and γ-globulins), hormones (cortisol and testosterone) and serum immunoglobulins (IgA, IgM, IgG). To assess the state of the antioxidant system, a quantitative analysis of the oxidative and antioxidant capacity of blood serum was carried out. The study was done using the ImAnOx (TAS/TAC) Kit and PerOx (TOS/TOC) Kit from Immundiagnostik. The analysis of the antioxidant capacity was based on the reaction of antioxidants in the sample with exogenous hydrogen peroxide. The remaining peroxide was determined photometrically using a microplate reader. Antioxidant capacity was calculated in μmol of peroxide. Low antioxidant capacity was less than 280 μmol/L; medium, 280–320 μmol/L; high, more than 320 μmol/L [58,59].

The assessment of oxidative stress was based on the measurement of peroxide levels by the reaction of peroxidase with peroxides and photometric determination of the colored product. The level of oxidative stress was assessed on the basis of the data proposed by the manufacturers of the reagent kits (peroxides less than 180 μmol—low oxidative stress, 180–310 μmol/L—medium stress, more than 310 μmol/L—high oxidative stress) [60].

Complete blood count was performed using the Sysmex XS-800 hematology analyzer. The protein level was determined by the biuret method using the ROKI-6T photometer and Olvex Diagnosticum reagents [61]. Protein fractions were measured using the UEF-01-Astra electrophoresis device and KliniTest-EF reagents manufactured by EKO Service. We used cellulose acetate membranes and a set of sera. The electrophoretogram was scanned on a densitometer [62,63].

In blood serum, the concentrations of iron, magnesium, potassium, calcium, inorganic phosphorus, sodium and chlorides were determined using Olvex Diagnosticum reagents. Tests were carried out on the CLIMA MC-15 biochemistry analyzer. The concentrations of iron and magnesium were determined by the colorimetric method without deproteinization using chromogens Nitro-PAPS and xylidyl blue [64,65]. The concentration of potassium was determined by the nephelometric method without deproteinization using tetraphenylborate [66]. The level of calcium was determined by means of ortho-cresolphthalein complexone [67]. Inorganic phosphorus in blood serum was determined by spectrophotometric method without deproteinization. This method is based on the ability of phosphates to form a phosphorus–molybdate complex with ammonium molybdate, the optical density of which is proportional to the concentration of inorganic phosphorus in the tested sample [68]. The sodium concentration was determined by the colorimetric method, using a precipitating reagent [69] and the level of chlorides by the colorimetric method using mercury thiocyanate [70].

The concentration of zinc and copper in blood serum was measured using the Quantum-II/2 atomic absorption spectrometer. To determine the contents of vitamins A, E (in blood serum) and B_2_ (in blood), the bioliquid analyzer Fluorat-02-ABLF-T was used.

The content of vitamin A was determined by measuring the fluorescence of retinol in a hexane extract of serum previously exposed to water and ethanol. The determined concentration range was 0.1–1.0 μg/cm^3^ [71]. Vitamin E was measured by the fluorescence of alpha-tocopherol in a hexane extract of serum previously exposed to water and ethanol. The determined concentration range was 2–15 μg/cm^3^ [71].

The level of vitamin B_1_ was measured based on the content of the decomposition product of pyruvic acid. An increase in the content of the decomposition indicates a decrease in the concentration of this vitamin. The method is based on the reaction of pyruvic acid with 2,4-diphenylhydrazine resulting in the formation of hydrazine, which acquires a brownish-red color in an alkaline environment. The intensity of the color is directly proportional to the concentration of pyruvic acid. The determined content of pyruvic acid was 2.5–20 μg/cm3 without sample dilution [72]. The determination of vitamin B_2_ in whole blood was carried out according to the method of performing measurements of the mass concentration of the vitamin according to Birch, Bessey and Lowry. Determined concentration range 0.1–1.0 μg/cm3 without sample dilution [73]. The method is based on the extraction of various forms of riboflavin from the blood by trichloroacetic acid, followed by acid hydrolysis of flavin adenine dinucleotide and fluorometric determination of the content of riboflavin.

In the FP, the content of vitamins A, E, B_1_ and B_2_ was measured using the same methods and equipment as in blood. The content of minerals was determined by atomic absorption spectrometry on the “Kvant-2AT” and “Kvant.Z-ETA”.

To measure hormones in the blood serum, the concentration of cortisol and testosterone was determined using an enzyme-linked immunosorbent assay. We used reagent kits produced by Vector Best and AccuBind ELISA Microwells kit.

The effectiveness of the FP in disease prevention was assessed based on clinical and laboratory data (indicators of the metabolic status, hematopoietic system, humoral immunity, antioxidant protection) as well as on the assessment of morbidity from the first incidence of seeking medical assistance by students of the treatment and control groups (during thirty-four weeks of observation from day 1 to day 238 (structure (%) and level (per 1000 people, ‰) in total and weekly). The morbidity data were obtained from the medical records of patients receiving outpatient medical treatment (form No. 025/y).

All data were processed using Statistica 6.1 software. The mean values and a standard error of the mean (M ± m) were determined. For parametric data, Student’s *t*-test determined the reliability of differences; in the case of nonparametric data, the Mann–Whitney and Wilcoxon tests were employed.

## 3. Results

### 3.1. Analysis of the Micronutrient Content in the Functional Product

As the laboratory analysis shows, the functional product had a high content of minerals and vitamins compared to regular food (Table 1). 

### 3.2. Analysis of Blood Samples

Blood tests were performed thrice: before the intake of the functional food (day 21), on day 15 of the treatment (a day after the last intake, day 37) and on day 45 of the treatment (day 67). At the end of the observation period, the level of copper in blood samples of the treatment group was 12.3% (*p* < 0.05) higher than that of the control group. There was an increase in the level of iron by 15.2% (*p* < 0.05 magnesium-by 6.3% (*p* < 0.05) and of phosphorus by 16.1% (*p* < 0.05). At the end of the observation, the level of iron was 20.8% above the initial (*p* < 0.05), of magnesium 34.2% (*p* < 0.05) and phosphorus 21.2% (*p* < 0.05). In the control group, on the contrary, there was a 22.6% decrease in the level of magnesium (*p* < 0.05) and a 10.1% decrease in phosphorus (*p* < 0.05). In both groups, calcium, potassium, sodium and chlorine remained within the reference boundaries. Both groups showed no reliable dynamics in the content of these minerals (*p* > 0.05) (Table 2).

The intake of the functional product contributed to an increase in the level of vitamins in blood: the concentration of vitamin A increased by 21.3% (*p* < 0.05) and vitamin B_2_ by 13.5% (*p* < 0.05). At the end of the observation, the content of vitamin A was above the initial level by 23.6% (*p* < 0.05), vitamin E by 16.9% (*p* < 0.05) and vitamin B_2_ by 28.3% (*p* < 0.05). Both groups showed a decrease in the content of vitamin B_1_, which was more significant in the control group—17.0% at the end of the observation and 10.7 after 45 days. In the control group, no significant changes in the concentrations of vitamins A and B_2_ were registered. The content of vitamin E both by the end of the product intake (*p* < 0.05) and by the end of the observation (*p* < 0.05) was 12.5% below the initial value (Table 3). This can be attributed to its sufficient quantity in the diet. In the control group, pre-treatment vitamin E concentration was below the reference boundary in 40.0% of students. After 15 days, this percentage increased to 90.0% (φ = 3.567) and remained at this level until the end of the observation (80.0%, φ = 2.672).

At the end of the trial period, the total protein in the blood samples of the treatment group was significantly higher (5.7%) than that in the control group (*p* < 0.05). The content of albumin in the treatment group did not change. In the control group at each stage of observation, it was above the initial value by 8.2% (*p* < 0.05) and 11.5% (*p* < 0.05), respectively. In the treatment group, a decrease in α-1 globulins was noted—by 12.4% (*p* < 0.05) and 14.6% (*p* < 0.05), respectively. In the control group, at the end of the observation period, there was a 15.5% (*p* < 0.05) decrease in α-2 globulins. In the treatment group, the figure did not change significantly. After a meal including the FP, β-globulins increased by 8.0% (*p* < 0.05), a change that was not observed in the control group. γ-globulins in the control group decreased significantly by the end of the trial period by 18.7% (*p* < 0.05) and by the end of the observation period by 19.0% (*p* < 0.05). In the treatment group, an 8.6% (*p* < 0.05) increase in this fraction of globulins was noted after a meal including the FP (Table 4). 

In the control group, testosterone had decreased by 29.6% (*p* < 0.05) by the end of the food intervention period and by 32.8% (*p* < 0.05) after another 30 days. In the treatment group, only at the end of the observation period was there a decrease of 14.3% in the level of this hormone. However, it was not significant (*p* > 0.05). Cortisol dynamics in both groups did not change significantly (Table 5).

Hemogram analysis showed changes in red and white blood cells and platelets, which were within the reference values. In both groups, red blood cells, erythrocytes, hemoglobin and hematocrit did not change significantly. The average erythrocyte volume in the control group increased by 1.1% (*p* < 0.05) and 1.3% (*p* < 0.05) at different stages of the observation (Table 6). On day 16, a 1.2% decrease (*p* < 0.05) in the hemoglobin content in the erythrocyte was noted. This level remained almost unchanged until the end of observation: there was a slight decrease of 0.8% (*p* < 0.05). The same dynamics was observed in the average concentration of hemoglobin in erythrocytes: there was a 2.0% (*p* < 0.05) and 2.8% (*p* < 0.05) decrease, respectively. In the treatment group, an increase in the relative *RDW* was noted: a standard deviation (by 2.5% (*p* < 0.05 after 15 days and *p* < 0.05 after 45 days) and the coefficient of variation (by 2.0 (*p* < 0.05) and 2.3% (*p* < 0.05) respectively).

Table 7 shows that white blood cells were affected more dramatically. In the treatment group, by the end of the course, the leukocyte content increased by 12.0% (*p* < 0.05) and remained at this level until the end of the observation period: the excess was 10.4% (*p* < 0.05). The growth of lymphocytes reached 9.4% (*p* < 0.05) and 8.6% (*p* < 0.05) at different stages of observation and monocytes by 14.5% (*p* < 0.05) and 21.7% (*p* < 0.05), respectively. In the control group, an increase in the number of lymphocytes was registered only after 45 days of observation and was 24.9% (*p* < 0.05). The relative content of lymphocytes in the observation groups did not change, and monocytes in the treatment group increased by 11.4% by the end of observation (*p* < 0.05). Neutrophils in the treatment group increased within the normal range by 17.3% (*p* < 0.05) by the end of the food intervention period. However, at the end of the observation, this value did not significantly differ from the initial (*p* < 0.05). There was also an increase in basophils within the normal range. By the end of the FP course, their value rose by 21.7% (*p* < 0.05) and by 30.0% (*p* < 0.05). In the control group, only the growth of lymphocytes was observed by the end of the observation.

In the treatment group, at the end of the treatment, there was a 4.8% (*p* < 0.05) increase in platelets and a 3.7%, (*p* < 0.05) decrease in the relative PDW. This effect did not persist until the end of the observation period (Table 8). Still, a decrease in the coefficient of large platelets by 5.3% (*p* < 0.05) was observed before the end of the study: 6.3% (*p* < 0.05). In the control group, a decrease in the coefficient of large platelets by 6.9% was registered only after 45 days into the observation (*p* < 0.05).

Initially, the presence of a high level of oxidative stress in the body of students in each subgroup was evidenced by the data on peroxide levels. In the treatment group, the level of peroxides decreased to a level estimated as the average: by the end of the FP intake, by 33.7% (*p* < 0.05) and after one month of observation by 62.8% (*p* < 0.05). No changes occurred in the control group (Table 9), but the antioxidant activity of the serum in this group increased by 24.9% by the end of observation (*p* < 0.05). At the beginning of observation and the end of the course, it was assessed as average, and 45 days later as high. In the control group, a decrease in the antioxidant activity of blood serum was observed, by 11.2% (*p* < 0.05) at the end of the FP course and by 24.4% (*p* < 0.05). By the end of observation, the average antioxidant activity decreased to a low value.

In the treatment group, the level of IgA during the observation period did not change significantly. In the control group, it decreased by 37.5% (*p* < 0.05) by the end of the observation. IgM in the treatment group increased only by the end of the observation (by 43.3%, *p* < 0.05). In the control group, an increase in IgM was registered after 15 days of observation (by 80.4%, *p* < 0.05). After 45 days, this value was 54.0% above the initial one (*p* < 0.05). IgG in the treatment group increased by 43.6% (*p* < 0.05) by the end of the observation, whereas in the control group, no significant changes occurred. 

### 3.3. Morbidity Pattern Analysis

The morbidity pattern in students in the control group was the same as in the comparison group (Table 10). Respiratory diseases and skin and subcutaneous tissue conditions were the first and second most widespread, respectively.

In the treatment group, the 34 weeks incidence rate was 0.6 times that in the control group (Table 11). There was a difference in the incidence rate of respiratory diseases (1.7 times less frequent) and skin and subcutaneous tissue conditions (5.7 times less frequent).

Among diseases of the respiratory system, two nosological forms demonstrated certain differences: acute viral respiratory infections of the upper respiratory tract (ARI URT) and influenza, as well as community-acquired pneumonia (CAP). The incidence of the former was 1.8 times lower in the treatment group than in the control group. There were no cases of CAP in the treatment group (Table 12).

**Figure 1:**
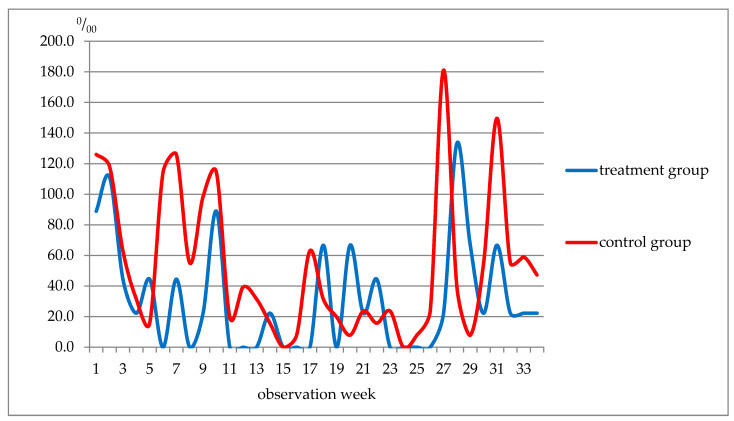
Weekly morbidity in students (respiratory system diseases), ‰.The data in Table 13 show that, although the incidence of respiratory diseases in the control group was slightly higher before and during the treatment period (weeks 1–5), the incidence of acute viral respiratory infections of the upper respiratory tract (ARI URT) and flu was almost the same in the two groups. Further, in weeks 6–10, these indicators in the treatment group were lower than in the control group, 3.3 times and 5.6 times, respectively. In weeks 11–19 of the observation, the incidence of respiratory diseases in the treatment group was 2.6 times lower and in weeks 23–27, there was a 10.6-fold decrease in the disease incidence rate. Such a shift was not noted for acute respiratory diseases and flu. It was not earlier than weeks 28–34 when the incidence of ARI, upper respiratory tract infection and influenza began to approach that in the control group (Figure 2).

The weekly analysis of diseases revealed several interesting patterns (Figure 1). For example, fewer respiratory diseases were reported in the control group only in weeks 20–22, whereas during the remaining weeks of the observation period, respiratory diseases were more often registered.

**Figure 3:**
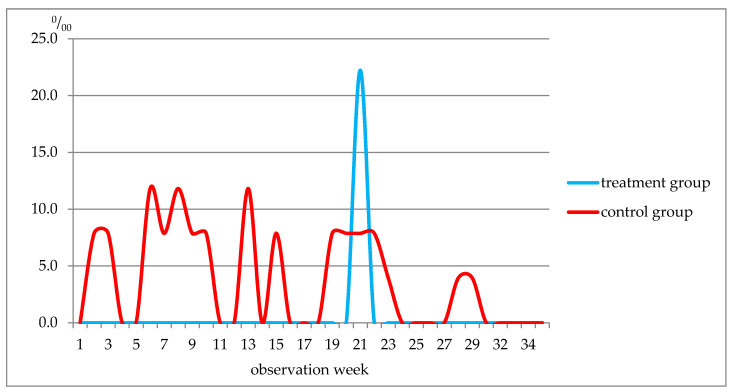
The incidence rate of diseases of the skin and subcutaneous tissue in the control group by observation weeks, ‰.

In the treatment group, diseases of the skin and subcutaneous tissue were diagnosed only in week 21, whereas in the control group, they were diagnosed in weeks 2–3, 6–10, 13, 15, 19–23, 28–29 (Figure 3). The incidence rate in the treatment group was 22.2‰, and in the control group, it was 126.0 ‰.

## 4. Discussion

Prior studies have noted that human health can be affected by a combination of factors, including the living environment [74,75,76,77]. Upon moving from across the country to the place of their study, students need to adapt to a new climatic and social environment [78]. Climatic and social adaptation is a stress factor affecting the adaptive potential of the body. This fact has been noted by many authors [79,80,81]. The results of our study are consistent with these findings: students in the control group experienced a number of negative changes in their body parameters during the period of their adaptation to the Baltic climate. Our study also suggests that the length of the adaptation period depends on the speed of acclimatization.

The presence of stress in students during the period of climatic and social adaptation was evidenced by increased levels of cortisol. These findings were in agreement with those obtained by other researchers who noted that the level of cortisol is directly linked to stress and anxiety that students experience during exams [82,83], as well as to chronic lack of sleep in adolescents [84], and the effects of psychological and social stress in mentally unwell patients [85]. Previous studies noted that a higher level of cortisol is found in people whose profession is associated with increased risk and/or permanent stress, particularly police officers [86] and medics [87]. An increased level of this hormone in humans may be a precursor of acute myocardial infarction [88,89]. In contrast, testosterone levels tend to decrease. This effect is also observed in the overtraining syndrome of athletes. This disorder is caused by excessive training combined with insufficient rehabilitation and poor quality of sleep, which leads to poor performance and fatigue [90]. This dynamic of cortisol and testosterone indicates the predominance of catabolic processes in nutrient metabolism as the body’s need for vitamins and minerals increases [91].

In our study, deviations in the values of albumin and each of the globulin fractions caused disorders in the body, which became more considerable with fatigue. An increase in the level of albumin may have been caused by dehydration. Researchers have had similar findings analyzing serum albumin in the blood plasma of athletes after intense training [92,93]. An increase in the values of α1- and α2-globulins, which include proteins of the acute phase, can result from stress and inflammatory reactions in the body. The same effect was observed following an insufficient intake of magnesium in food [94,95]. 

The count of red blood cells testified to a negative impact of the change in the climatic and social environment during the adaption period: there was a decrease in the hemoglobin content and in the average concentration of hemoglobin in erythrocytes. The compensatory reaction of the body manifested in an increase in the average volume of erythrocytes. Researchers studying the adaptation of people to the Arctic climate of Russia’s North have noted similar changes [96]. 

In our study, a decrease in humoral immunity was evidenced by the dynamics of β- and γ-globulins in blood serum and serum IgA. After 15 days of observation, an inflammatory reaction was observed by an increase in IgM. The immune response was insufficient: no IgG growth was recorded after 45 days of observation. A high level of oxidative stress and an increase in the low antioxidant capacity of serum were observed. This is consistent with other research noting a similar effect characteristic of the chronic fatigue syndrome, which can lead to changes in the endocrine function, as well as to immune-related diseases, systemic inflammations and other health-threatening conditions [97].

Based on the scientific evidence available, the European Food Safety Authority (EFSA) considers six vitamins (D, A, C, folic acid, B_6_ and B_12_) and four minerals (zinc, iron, copper and selenium) essential for the normal functioning of the immune system [98]. Vitamins A to E are known to be beneficial in treating COVID-19 since they have antioxidant and immune-stimulatory properties [99].

The treatment group demonstrated an increase in the levels of vitamins (A, E and B_2_; vitamin B_1_ decreased slightly) and minerals (copper, zinc, iron, magnesium and phosphorus) in their blood. The level of testosterone did not go down, and there was no increase in catabolic reactions. This can probably be explained by the positive effects of the food intervention on the hematopoietic system. In particular, the average volume of erythrocytes grew more slowly than in the control group, the average concentration and content of hemoglobin did not decrease and the relative RDW went up. The count of white blood cells showed that the immune system positively responded to the food intervention, which reflected in the number of lymphocytes, the relative content of neutrophils and an increase in the number of monocytes, basophils and eosinophils. Protein metabolism indicators evidenced a positive effect of the FP on humoral immunity: an increase in the concentration of β-globulins (including complements involved in the immune response) and an increase in γ-globulins. Increased resistance of the body was confirmed by an increase in IgM at a later stage as well as an increase in IgG as an indicator of an active immune response. Signs of oxidative stress considerably weakened. 

A strong relationship between a poor nutritional status and the risk of severe respiratory diseases, including COVID-19, has been reported in the literature [100]. Our findings, while preliminary, confirmed the effectiveness of the food intervention, which manifested in a lower incidence of respiratory diseases as well as skin and subcutaneous tissue conditions in the treatment group. Weekly analyses showed that after the end of the FP course (week 6) to week 27 of the observation, respiratory diseases were more frequent in the control group. Among the diseases of the respiratory system, a consistent downward tendency in the frequency of occurrence of acute viral respiratory infection of the upper respiratory tract and flu and community-acquired pneumonia was observed. The effectiveness of the proposed method was also proven by a decrease in the incidence of skin and subcutaneous tissue diseases.

The study helped reveal the impact of risk factors associated with adaptation and acclimatization during the first weeks at university in regions having specific weather and climatic conditions. It also made it possible to assess the effectiveness of preventive measures for the protection of students’ health.

## 5. Conclusions 

The present research aimed to assess the effectiveness of the proposed food intervention in preventing the most common diseases among students who relocate to the Kaliningrad region from other regions of Russia. Our analysis showed that during the period of adaptation and acclimatization, catabolic processes prevailed in the metabolism of nutrients. The study of protein metabolism, vitamin and mineral concentrations, hematopoiesis and humoral immunity revealed disadaptation changes in the body. The weakness of immune response, α1- and α2-globulins and the levels of IgM and IgG confirmed the presence of inflammatory reactions. Oxidative stress was analyzed by measuring the level of peroxides and the anti-oxidative capacity of serum. After enriching the diet of the treatment group with the functional food, a decrease in testosterone was no longer observed, thus preventing an increase in nutrient catabolism. A positive effect on hematopoiesis was registered. In red blood cells, the average volume of erythrocytes grew less rapidly. The average concentration and the content of hemoglobin did not decrease. The relative range of the distribution of erythrocytes by volume increased. Our findings suggest that the immune system can positively respond to the food intervention (lymphocytes, the relative content of neutrophils and an increase in the number of monocytes, basophils and eosinophils). The stimulation of humoral immunity was confirmed by an increase in beta and gamma globulins. An active immune response by IgM and IgG to inflammation was observed. According to the obtained data, the antioxidant capacity increased. 

The study showed that after 34 weeks of the FP intervention, the incidence of diseases was lower in the treatment group compared with the control group (the incidence of respiratory diseases decreased 1.7-fold, and that of skin and subcutaneous tissue conditions 5.7-fold). There was a decrease in the incidence of ARIs of URT, which dropped 1.8-fold. There were no cases of community-acquired pneumonia (versus 55.1‰ in the control group). In weeks 6–19, the incidence of respiratory diseases in the treatment group was 3.3–10.6 times lower than in the control group.

Further research needs to be done into the potential of different types and compositions of functional food for preventing specific diseases.

## Figures and Tables

**Figure 2:**
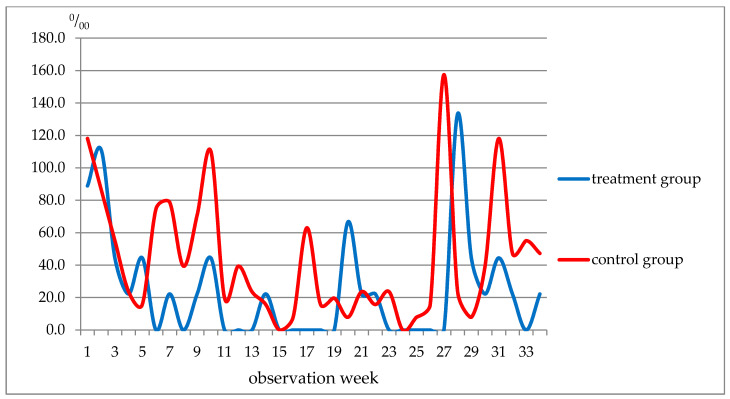
The incidence of ARI URT and flu among students of the control group by observation weeks, ‰.

**Table 1:** The vitamin and mineral content in the functional product, mg/100 g.

Minerals					Vitamins				
Cu	Zn	Fe	Мn	Cr	A	E	В_2_	K	C
0.58	9.86	71.00	2.85	0.16	0.03	3.69	0.30	0.51	89.91

**Table 2:** Body mineral concentrations during the observation period (absolute values).

Parameter (Reference Values)	Observation Period, М ± m		
	Start (21 Day)	End (37 Day)	45 Days Later (67 Day)
Minerals			
Copper, μg/mL (0.7–1.55):			
treatment	0.85 ± 0.03	0.82 ± 0.06 (−3.6%)	0.82 ± 0.03 (−3.6%)
control	0.81 ± 0.09	0.73 ± 0.05 (−9.9%)	0.76 ± 0.04 (−6.2%)
Zinc, μg/mL (0.5–1.5):			
treatment	1.27 ± 0.10	1.20 ± 0.07 (−5.5%)	1.30 ± 0.05 (+2.3%)
control	1.0 ± 0.08	0.97 ± 0.08 (−3.0%)	1.13 ± 0.08 (+13.0%)
Iron, mM/L (11.6–31.3):			
treatment	13.60 ± 0.73	15.67 ± 1.0 (+15.2%)	16.43 ± 1.0 (+20.8%)
control	14.20 ± 1.15	16.84 ± 0.86 (+18.5%)	16.65 ± 0.93 (+17.2%)
Magnesium, mM/L (0.66–1.07):			
treatment	0.63 ± 0.01	0.67 ± 0.01 (+6.3%)	0.85 ± 0.01 (+34.2%)
control	0.99 ± 0.02	0.77 ± 0.01 (−22.6%)	0.86 ± 0.01 (−13.6%)
Phosphorus, mM/L (0.87–1.45):			
treatment	1.12 ± 0.03	1.29 ± 0.03 (+16.1%)	1.35 ± 0.03 (+21.2%)
control	1.26 ± 0.03	1.13 ± 0.03 (−10.1%)	1.30 ± 0.03 (+3.4%)
Calcium, mM/L (2,15–2,57):			
treatment	2.61 ± 0.02	2.59 ± 0.01 (−0.8%)	2.58 ± 0.02 (−1.1%)
control	2.56 ± 0.02	2.51 ± 0.02 (−1.9%)	2.54 ± 0.20 (−0.8%)
Potassium, mM/L (3,6–5,5):			
treatment	5.19 ± 0.12	5.04 ± 0.10 (−2.9%)	5.25 ± 0.11(+1.1%)
control	5.31 ± 0.15	5.10 ± 0.08 (−4.0%)	5.17 ± 0.09 (−2.7%)
Sodium, mM/L (135–150):			
treatment	145.60 ± 2.31	149.60 ± 0.35 (+2.7%)	150.50 ± 0.48 (+3.3%)
control	149.80 ± 0.57	147.60 ± 0.36 (−1.5%)	150.0 ± 0.38 (+0.1%)
Chlorine, mM/L (97–108):			
treatment	100.20 ± 1.11	100.30 ± 0.40 (+0.1%)	104.30 ± 0.40 (+4.1%)
control	102.10 ± 0.47	103.40 ± 0.48 (+1.3%)	102.80 ± 0.41 (−0.9%)

**Table 3:** Vitamin concentrations during the observation (absolute values).

Parameter (Reference Values)	Observation Period, М ± m		
	Start (21 Day)	End (37 Day)	45 Days Later (67 Day)
Vitamins			
A, μg/mL (0.3–0.6):			
treatment	0.89 ± 0.04	1.08 ± 0.04 (+21.3%)	1.1 ± 0.03 (+23.6%)
control	0.83 ± 0.05	0.91 ± 0.05 (+9.6%)	0.91 ± 0.04 (+9.6%)
E, μg/mL (8–18):			
treatment	7.86 ± 0.34	8.7 ± 0.54 (+10.7%)	9.19 ± 0.41 (+16.9%)
control	7.75 ± 0.43	6.78 ± 0.39 (−12.5%)	6.78 ± 0.53 (−12.5%)
В1, μg/mL (7–14):			
treatment	21.4 ± 0.9	20.6 ± 1.0 (−3.8%)	20.63 ± 1.0 (−3.6%)
control	19.7 ± 1.3	16.36 ± 0.85 (−17.0%)	17.6 ± 1.0 (−10.7%)
B2, μg/% (10–50):			
treatment	6.1 ± 0.03	6.9 ± 0.41 (+13.5%)	7.83 ± 0.2 (+28.3%)
control	5.91 ± 0.4	5.85 ± 0.3 (−0.9%)	6.08 ± 0.3 (+2.8%)

**Table 4:** Protein metabolism indicators during the observation period (absolute values).

Parameter (Reference Values)	Observation Period, М ± m		
	Start (21 Day)	End (37 Day)	45 Days Later (67 Day)
Protein metabolism			
Total protein, g/L (64–83):			
treatment	74.15 ± 0.68	75.9 ± 1.0 (+2.3%)	74.6 ± 0.8 (+0.6%)
control	73.2 ± 0.60	71.8 ± 0.7 (−1.9%)	74.8 ± 0.9 (+2.1%)
Albumin,% (46.9–61.4):			
treatment	55.71 ± 0.7	54.29 ± 0.64 (−2.6%)	55.44 ± 0.88 (−0.5%)
control	51.95 ± 1.0	56.23 ± 0.8 (+8.2%)	57.91 ± 0.74 (+11.5%)
Alpha−1 globulins,% (2.2–4.2):			
treatment	4.37 ± 0.17	3.83 ± 0.1 (−12,4%)	3.73 ± 0.14 (−14.6%)
control	4.21 ± 0.13	4.05 ± 0.2 (−3,8%)	4.24 ± 0.71 (+0.7%)
Alpha−2 globulins,% (7.9–10.9):			
treatment	10.2 ± 0.23	9.66 ± 0.25 (−5.3%)	10.02 ± 0.49 (−1.8%)
control	11.68 ± 0.44	10.06 ± 0.3 (−13.9%)	9.91 ± 0.54 (−15.5%)
Beta globulins,% (10.2–18.3):			
treatment	11.4 ± 0.26	12.31 ± 0.23 (+8.0%)	11.61 ± 0.42 (+1.8%)
control	11.79 ± 0.29	12.13 ± 0.18 (+2.8%)	11.3 ± 0.52 (−4.2%)
Gamma globulins,% (17.6–25.4):			
treatment	18.28 ± 0.53	19.85 ± 0.58 (+8.6%)	19.19 ± 0.85 (+4.9%)
control	20.54 ± 0.8	16.69 ± 0.73 (−18.7%)	16.63 ± 0.59 (−19.0%)

**Table 5:** Changes in the hormone content during the observation period (nmol/L).

Parameter (Reference Values)	Observation Period, М ± m		
	Start (21 Day)	End (37 Day)	45 Days Later (67 Day)
Hormones, (nmol/L)			
Testosterone, 8.72–38.17:			
treatment	24.73 ± 1.98	25.53 ± 1.65 (+3.2%)	21.19 ± 1.77 (−14.3%)
control	26.18 ± 1.17	18.43 ± 1.8 (−29.6%)	17.59 ± 0.9 (−32.8%)
Cortisol, 200.0–700.0:			
treatment	636.8 ± 31.8	673.3 ± 29.6 (+5.8%)	672.0 ± 35.0 (+5.5%)
control	750.6 ± 47.9	761.0 ± 38.4 (+1.4%)	685.6 ± 28.5 (−8.7%)

**Table 6:** Red blood cells during the period of food intervention (absolute values).

Parameter (Reference Values)	Observation Period, М ± m		
	Start (21 Day)	End (37 Day)	45 Days Later (67 Day)
Average erythrocyte volume, 80–95 fl:			
treatment	87.1 ± 0.49	87.5 ± 0.47 (+0.5%)	88.3 ± 0.54 (+1.3%)
control	85.1 ± 0.9	86.0 ± 0.99 (+1.1%)	86.2 ± 0.9 (+1.3%)
Average content of HGB in erythrocyte, 25–35 pg:			
treatment	30.30 ± 0.17	30.35 ± 0.17 (+0.1%)	30.49 ± 0.17 (+0.6%)
control	30.35 ± 0.39	30.0 ± 0.37 (−1.2%)	30.1 ± 0.23 (−0.8%)
Average concentration of HGB in erythrocyte, 30.0–38.0 g/L:			
treatment	34.79 ± 0.08	34.67 ± 0.14 (+0.4%)	34.61 ± 0.20 (−0.5%)
control	35.6 ± 0.3	34.9 ± 0.19 (−2.0%)	34.6 ± 0.24 (−2.8%)
Relative RDW, standard deviation,			
39–46 fl.:			
treatment	41.45 ± 0.33	42.5 ± 0.43 (+2.5%)	42.48 ± 0.35 (+2.5%)
control	42.07 ± 0.5	42.39 ± 0.68 (+0.6%)	42.5 ± 0.69 (+1.0%)
Relative RDW, coefficient of variation,			
11.8–15.6%:			
treatment	13.28 ± 0.10	13.54 ± 0.12 (+2.0%)	13.59 ± 0.10 (2.3%)
control	13.2 ± 0.09	13.2 ± 0.10 (0%)	13.1 ± 0.13 (−0.8%)

**Table 7:** White blood cells during the food intervention (absolute values).

Parameter (Reference Values)	Observation Period, М ± m		
	Start (21 Day)	End (37 Day)	45 Days Later (67 Day)
Leukocytes, 4.2–9 × 10^9^ cells/L:			
treatment	6.96 ± 0.22	7.86 ± 0.30 (+12.0%)	7.69 ± 0.21 (+10.4%)
control	6.57 ± 0.48	6.31 ± 0.34 (−3.9%)	7.95 ± 0.35 (+24.9%)
Lymphocytes, 1.5–4.0 × 10^9^ L:			
treatment	2.33 ± 0.07	2.55 ± 0.09 (+9.4%)	2.53 ± 0.10 (+8.6%)
control	2.17 ± 0.16	2.23 ± 0.18 (+2.7%)	2.71 ± 0.17(+24.9%)
Monocytes, 0.1–0.8 × 10^9^ L:			
treatment	0.69 ± 0.03	0.79 ± 0.04(+14.5%)	0.84 ± 0.03(+21.7%)
control	0.6 ± 0.023	0.62 ± 0.03 (+3.3%)	0.63 ± 0.03 (+5.0%)
Relative content of monocytes, 2–11%:			
treatment	9.87±0.31	9.94±0.30 (+0.7%)	11.0±0.46 (+11.4%)
control	9.1±0.23	9.16±0.3 (+0.6%)	9.2±0.33 (1.0%)
Neutrophils, 2.0–7.7 × 10^9^ L:			
treatment	3.58 ± 0.19	4.2 ± 0.26 (+17.3%)	4.0 ± 0.19 (+11.7%)
control	4.24 ± 0.34	3.80 ± 0.22 (−10.4%)	4.11 ± 0.34 (−3.1%)
The relative content of neutrophils, 42–72%:			
treatment	50.7 ± 1.2	51.47 ± 1.4 (+1.5%)	51.63 ± 1.54 (+1.8%)
control	54.5 ± 1.86	53.2 ± 2.3 (−4.3%)	52.67 ± 1.9 (−3.4%)
Basophil content, 0.02–0.1 × 10^9^ L:			
treatment	0.02 ± 0.001	0.024 ± 0.002 (+21.7%)	0.026 ± 0.001 (+30.0%)
control	0.02 ± 0.001	0.021 ± 0.001 (+5.0%)	0.02 ± 0.001 (0%)

**Table 8:** Platelets during the food intervention (absolute values)

Parameter (Reference Values)	Observation Period, М ± m		
	Start (21 Day)	End (37 Day)	45 Days Later (67 Day)
Platelets, 180–400 × 10^9^ cells/L:			
treatment	231.8 ± 6.53	242.97 ± 6.9 (+4.8%)	243.9 ± 8.5 (+5.6%)
control	221.1 ± 11.2	229.8 ± 9.35 (+3.9%)	240.2 ± 11.4 (+8.6%)
Relative PDW, 15–17%:			
treatment	13.55 ± 0.26	13.05 ± 0.25 (−3.7%)	12.84 ± 0.23 (−5.3%)
control	13.97 ± 0.7	13.78 ± 0.8 (−1.4%)	14.35 ± 0.9 (+2.7%)
P−LCR, 13–43%:			
treatment	34.29 ± 1.02	32.49 ± 1.05 (−5.3%)	32.12 ± 1.00 (−6.3%)
control	32.15 ± 3.0	29.99 ± 3.1 (−6.8%)	29.94 ± 2.9 (−6.9%)

**Table 9:** Antioxidant defense system and serum immunoglobulins during the period of food intervention (absolute values).

Parameter (Reference Values)	Observation Period, М ± m		
	Start (21 Day)	End (37 Day)	45 Days Later (67 Day)
Antioxidant protection system, μmol/L			
Peroxides, ˂180.0:			
treatment	542.3 ± 65.8	359.3 ± 58.5 (−33.7%)	201.7 ± 49.8 (−62.8%)
control	535.4 ± 49.8	519.5 ± 70.3 (−3.0%)	485.4 ± 59.8 (−9.4%)
Serum antioxidant activity, 280 ± 20.5:			
treatment	313.9 ± 9.8	313.5 ± 13.3 (−0.1%)	392.2 ± 11.3 (+24.9%)
control	311.5 ± 11.3	276.7 ± 9.5 (−11.2%)	235.4 ± 8.5 (−24.4%)
Serum immunoglobulins (g/L)			
IgA, 0.9–4.5:			
treatment	0.938 ± 0.1	1.034 ± 0.12 (+10.2%)	0.902 ± 0.11 (−3.9%)
control	0.685 ± 0.09	0.74 ± 0.09 (+8.0%)	0.428 ± 0.03 (−37.5%)
IgM, 0.6–3.7:			
treatment	1.258 ± 0.14	1.176 ± 0.14 (−6.6%)	1.803 ± 0.11 (+43.3%)
control	0.87 ± 0.07	1.57 ± 0.36 (+80.4%)	1.34 ± 0.16 (+54.0%)
IgG, 8–17:			
treatment	10.82 ± 1.19	11.98 ± 1.28 (+10.7%)	15.54 ± 1.39 (+43.6%)
control	15.92 ± 1.02	15.46 ± 1.39 (−3.1%)	11.49 ± 1.8 (−27.8%)

**Table 10:** Morbidity patterns in the control group by the most prevalent classes of diseases, %, rank.

Disease Class	Morbidity Pattern			
	Treatment		Control	
	%	Rank	%	Rank
VI. Diseases of the nervous system	1.3	4	1.0	4
X. Respiratory diseases	88.5	1	88.3	1
XI. Diseases of the digestive system	0.7	6	0.7	6
XII. Diseases of the skin and subcutaneous tissue	6.3	2	6.3	2
ХIII. Diseases of the musculoskeletal system and connective tissue	1.0	5	1.0	5
XIX. Injury, poisoning and some other consequences of exposure to external causes	2.2	3	2.2	3

**Table 11:** The distribution of diseases by the most prevalent classes during the observation period, ‰.

Disease Class	Disease Distribution, М ± m	
	Treatment	Control
VI. Diseases of the nervous system	22.2 ± 21.9	15.7 ± 7.8
X. Respiratory diseases	22.2 ± 21.9	15.7 ± 7.8
XI. Diseases of the digestive system	1066.6 ± 39.7	1788.4 ± 74.4
XII. Diseases of the skin and subcutaneous tissue	22.2 ± 21.9	125.9 ± 20.8
ХIII. Diseases of the musculoskeletal system and connective tissue	22.2 ± 21.9	15.7 ± 7.8
XIX. Injury, poisoning and some other consequences of exposure to external causes	66.6 ± 37.2	74.8 ± 16.5
Total	1222.2 ± 77.7	2062.9 ± 92.9

**Table 12:** Primary incidence of diseases of the respiratory system, М±m.

Disease Class	Morbidity, ‰	
	Treatment	Control
Acute sinusitis	44.4 ± 30.7	78.7 ± 16.9
Acute tonsillitis	177.7 ± 56.9	141.7 ± 21.8
Acute viral respiratory infection of the upper respiratory tract, flu	822.2 ± 56.9	1468.5 ± 52.0
Community-acquired pneumonia	0.0	55.1
Acute viral respiratory infection of the lower respiratory tract	22.2 ± 21.9	35.4 ± 11.6

**Table 13:** Morbidity patterns in the control group by the most prevalent disease classes,%, rank.

Week of Observation	Respiratory Diseases, Total		ARI of URT and Flu Included	
	Treatment	Control	Treatment	Control
1–5	311.0	354.3	314.0	299.1
6–10	155.5	507.9	66.7	374.0
11–19	88.9	228.4	0	204.7
20–22	66.7	47.1	66.7	47.2
23–27	22.2	235.6	0	204.7
28–34	288.7	413.4	288.7	339.5
